# Continuous integration in urban social-ecological systems science needs to allow for spacing co-existence: *This article belongs to* Ambio*’s 50th Anniversary Collection. Theme: Urbanization*

**DOI:** 10.1007/s13280-020-01449-y

**Published:** 2021-03-12

**Authors:** Dagmar Haase

**Affiliations:** 1 Department of Geography, Humboldt Universität zu Berlin, Rudower Chaussee 16, 12489 Berlin, Germany; 2 Department of Computational Landscape Ecology, Helmholtz Centre for Environmental Research, Permsoser Street 15, 04318 Leipzig, Germany

**Keywords:** Integration, Social-ecological systems, Urban systems

## Abstract

Urbanization brings benefits and burdens to both humans and nature. Cities are key systems for integrated social-ecological research and the interdisciplinary journal of *Ambio* has published ground-breaking contributions in this field. This reflection piece identifies and discusses integration of the human and natural spheres in urban social-ecological research using the following foundational papers as important milestones: Folke et al. (1997), Ernstson et al. (2010) and Andersson et al. (2014). These papers each take unique approaches that aim to uncover core properties—processes, structures, and actors—of urban systems and set them into mutual relationship. This piece will end with a forward-looking vision for the coming 50 years of urban sustainability and resilience study in *Ambio*.

## My reflection

Cities are hubs of invention and innovation, places of productive conflict and a multitude of emergent phenomena in the behaviour of living systems and their complementary economic and social agent’s decision-making (Batty 2013). However, cities thrive at the cost of their co-evolutionary partner: nature (Kühn and Klotz 2006; McDonald et al. 2019). Cities and nature are trapped into an unfortunate paradox: they are neighbours, and, at the same time, they destroy each other as they are interdependent but follow different paradigms (Haase 2014). In community ecology, we have a concept for such relation: parasitism. However, as humans we very much believe in and need to aim for another concept when thinking about human-environmental interactions in cities: mutualism or, alternatively, commensalism.

When we look back at the long development of science, we see—temporally aligned with the industrial revolution in the middle of the 19th century—a new standpoint starting gain attraction and substance in defiance of the divide between the humanities, social sciences and natural sciences (Barrows 1923). After the emergence of urban and human ecology in the 1970s and 1980s of the 20th century, nicely visualized in the form of a family tree by the great landscape ecologist Hartmut Leser (2008), the introduction of the cornerstone concept of ecosystem services a decade later (Costanza et al. 1997), most notably scientific papers published by *Ambio* and others illuminated a novel understanding of the above-mentioned dependence between cities and nature—something that I call their “imperative dilemma”. The dilemma lies in the biased relationship of urban society to nature as ecosystems are needed but polluted or eliminated at the same time. Urban planning is dealing with enormous trade-offs resulting from this dilemma every day (Jenks and Jones 2010).

The following key papers will comprehensively, but not exclusively, serve to illustrate how the concepts of resource sharing and the “co-existence” character of ecological and social systems in and around cities arose (in order of their appearance). These co-existence ideas appeared first with Folke et al. (1997), second with Ernstson et al. (2010), and third, with Andersson et al. (2014). In the following, these *Ambio* papers will be discussed and positioned relative to the broader picture of social-ecological, human-environmental science or, in simple terms following Barrows (1923), integrated geography. This will be followed by an overview of where the journey might take us.

The first paper under discussion, Folke et al. (1997), is fundamentally about the integration of the human sphere into the nature/ecosphere in cities. This paper tells us about the portion of ecosystem services appropriation that is explicitly driven by cities and their residents. That said, the authors do not explicitly address residents or civil society, which are handled as quasi-homogeneous. In the 1990s, alongside the rise of ecosystem services thinking, Folke et al. took an enormous step towards showing *why* ecosystem knowledge—structure and functional—is required in planning policy to make urban systems work more sustainably. Their idea to mainstream their results about basin-wide ecosystem appropriation with planners goes towards what we call co-development—a collective and cooperative process of different groups—today. Still, particularly when looking back from 2020, the 1997 paper seems clearly limited since it did not explicitly deal with the internal diversity or complexity of the urban population of individual cities. In 1997, however, this was the prevailing way to speak about cities as quasi-homogeneous bodies that can “act”; not only in ecology but also in many other sciences. Today, after almost 30 years of further communication and integration with social sciences, we as urban scholars still feel challenged by the high complexity of urban societies. For example, when reading the following paragraph of Folke et al. (1997), the authors already anticipated that complexity would be an intrinsic property of the human system in cities: “Clearly, there is a pressing need for improved governance to cope with such acute problems to maintain cities as centres for knowledge, culture, creativity and innovation” (p. 171). Moreover, the diversity recognized for the biological system is somehow overlooked for the human urban system when speaking about them as “cities”. The 1997 *Ambio* publication, in this sense, is a kind of top-down resilience thinking approach that treats cities as homogeneous wholes.

The second paper by Ernstson et al. (2010) develops the ideas discussed in Folke et al. and, after 12 years of increasingly integrative science, focuses on the properties of individuals, specific thresholds, and cross-scale interactions of urban social-ecological systems. The authors of this 2010 *Ambio* paper expand the scale at which urban resilience has been discussed thus far by integrating the core idea from the aforementioned integrated geography (Barrows 1923) that cities form both a part of a “system of cities” and a complex but diverse organism in their own right. Ernstson et al. argue that cities cannot be understood as container entities but should rather be thought of as diverse bodies that consist of many different conflicting individuals. Urban resilience is understood as a multi-layered and multifunctional ability to withstand different kinds of environmental or social/societal disturbances highlighted in one of the core sentences and insights of the paper here: “The city can be thought of as an agglomeration of contested spaces that generate a range of urban services, from transport, housing, and medical aid, to jobs and financial markets. A presumption in this article is that such services are inextricably linked to ecological processes and the focus lies on such ‘ecosystem services’” (p. 2). The *four moves*, in other words the contribution of geography on the “…most challenging issues of contemporary urbanization…” (p. 22), are (1) slow variables and thresholds, (2) cross-scale interactions between social and technological networks, (3) urban innovative capacity, and (4) harnessing urban innovation across scales. These moves provide a brilliant add-on approach to what Folke et al. suggested, i.e. the policy link. However, these moves also expand on social issues (e.g. housing segregation pattern, age or ethnic groups), actor networks, expert stakeholders, innovation hubs including the technological/technical sphere of cities (sensu McPhearson et al. 2016). What Ernstson et al. explicitly not do is broadly expand on the idea that cities are systems of dissimilar distributions of benefits and beneficiaries of ecosystem services, on the one hand, and systems of active civil engagement and participation, on the other.

In 2011, Folke et al. again published a paper that dealt with social-ecological resilience and humanity as major driving forces: “Human development and progress must be reconnected to the capacity of the biosphere and essential ecosystem services to be sustained.” (p. 719). The author team highlighted the idea that human stewardship could both initiate and facilitate social-ecological transformation at a planetary level. They anticipate an ongoing mind shift in urban societies of the 21st century towards a “collaboration with the biosphere”. Ernstson et al. (2010, p. 12) propose a more ambivalent notion that is open in all directions. They state: “Furthermore, since urban innovation is a driver of urbanization, it influences (often negatively) ecosystems across the globe, which places urban innovation at the heart of resilience for our ecosystems”.

The third paper, Andersson et al. (2014), arrived about five years after Ernstson et al. (2010) and 3 years after Folke et al. (2011). This paper exemplifies a period of prolific publication on the topic of urban sustainability and resilience. By synthesizing social-ecological research in the Stockholm region, it takes up ideas from all forerunners, 1997, 2010 and 2011 and dives deeply into the idea of reconnecting cities to the biosphere. Opposite to the intrinsically ecological piece by Folke et al. (1997), this paper focuses more on the human side of the urban social-ecological system, more specifically, the behavioural aspects of urban residents. Andersson et al. present the social-ecological urban system as something that “spans from investigating ecosystem properties to the social frameworks and personal values that drive and shape human interactions with nature” (p. 445). What is more, the idea of human stewardship takes centre stage in their argument when they address the planning and governance of urban green–blue infrastructure: “local stewards are critically important.” Individuals are identified and acknowledged as key drivers and core shapers of urban resilience in terms of their active roles, activities, and networks.

Reading all three papers, one becomes aware of an “evolution” of the understanding of human governance in 25 years of urban *Ambio* history. Governance as such is addressed by Folke et al. (1997) in a “silo-like entity” or black box manner. It is then identified as an important part of the urban system by Ernstson et al. (2010) and acknowledged as a “facilitator” of the urban landscape and local ecosystem services by “institutional designs and social movements” (p. 448). Andersson et al. understand the governance of the social-ecological system as civil society and as an ensemble of individuals and accept these individuals as a rational and emotional part of the urban system that has “opportunity […] to have meaningful interaction and provide stewardship of […] local ecosystems” (p. 448). Such ideas of an active reconnecting of cities with their (surrounding and in situ) ecosystems were taken up by a researcher consortia of large international projects such as URBES (biodiversa), GREENSURGE (EU FP7), CONNECTING Nature (EU H2020), ENABLE (biodiversa), and similar projects outside of Europe.

The discussed articles published in *Ambio* from 1997 to 2014 reflect the progress in integrative thinking and an awareness of possibly conflicting individualism when dealing with and disentangling urban systems—namely cross-purpose processes due to contradictory actions, for example when increasing efforts in greening cities go along with increasing numbers of private cars and respective air pollution. The papers portray a pioneering way of analysing and understanding cities as part of the global ecosphere as well as individual bodies where human (social and technological) system and ecosystem co-evolution emerges, occurs and changes (see McPhearson et al. 2016). These three papers have been referred to by many pioneering papers written after 2015 and form part of our basic understanding of the role of ecology *in* cities and *of* cities themselves. All three papers seek thematic as well as methodological integration as keys to the study of urban resilience.

Unsurprisingly, after the publication of each innovative paper, there is always progress. Reflection and re-thinking occur when applying concepts and testing them in real case studies. Key papers such as the three discussed here found their way into practice and face a conflicting—rich versus poor, white versus black—and paradoxical—greening leading to gentrification that pushes out those assumed to benefit from more green—urban world. Compared to the thoroughly positive understanding of urban complexity in the three discussed articles, many subsequent papers understand urbanization and thus cities, as an aggressive multiplier. In times of globalization, more specifically, as the facilitator of pathogens spreads across the (urban) world (Wu et al. 2017, in *Ambio*). Wu et al. critically acknowledge the enormous power of predisposing (dissimilar) socioecological conditions in and of cities—fully in line with Ernstson et al.—but, in Wu and others’ case, related to the destructive power of urban expansion into wild nature coming into touch with wild disease reservoirs. This thought leads to the current situation most cities are being confronted with that has been caused by the Sars-COV2 virus in the COVID-19 pandemic and its consequences on e.g. the labour market and educational progress. In addition, we see protests against injustice and unfair living conditions in many cities worldwide.

## A vision for the coming 50 years of urban sustainability and resilience study in *Ambio*

The latter reflection brings me now to my vision for future publications on sustainability and resilience of social-ecological urban systems in *Ambio*. This vision starts from the three outstanding *Ambio* papers (Folke et al. 1997; Ernstson et al. 2010; Andersson et al. 2014) acknowledges (Folke et al. 2011; McPhearson et al. 2016; Wu et al. 2017) but expands upon the following crucial points:

1. Cities are hotspots of today’s crises; they are risky spots characterised by many different vulnerabilities (Bai et al. 2011, 2014), be it the recent COVID-19 pandemic, heat waves or coastal and river flooding. Cities are densely populated and most of them face further densification (Haase et al. 2013; Wolff and Haase 2019). Cities are hubs of mobility and interaction, as Wu et al. have already pointed out, and they are especially likely to become laboratories of (pandemic) crises, as we are now experiencing in e.g. Paris, Madrid, urban Lombardy, New York City, and elsewhere. At the same time, since the pandemics and climate crises of today and tomorrow will always also be urban crises, the civil societies of cities need to deal with them. Dealing with current crises means critically reflecting on both the study and understanding of social-ecological systems, with the eyes of an urban resident as well as being a part of urban nature. What I found in all three exciting papers is that―somehow―urban ecosystems are social-ecological systems in the way that they follow biophysical rules as well as governance ideas seemingly independent from deep-routed decisive rifts and imbalances in our urban societies.
2. A great potential for a richer, more inclusive discussion about transition, compared to normative, tipping point and threshold-based approaches, can be possibly found in ideas that actively include multiple and controversial outcomes such as biocultural diversity (Elands et al. 2018) or hybridity (Pieterse 1994). Both concepts understand themselves as disciplinary and concept-‘bridging’ frameworks. The current COVID-19 crisis also sheds light on existing inequalities and injustices within our urban societies and in terms of how people can adapt to and cope with restrictions (Haase 2020). It is much easier to endure restrictions in a large flat with balcony, garden or rooftop access and that is close to green spaces than in a small flat packed with people. Lack of easy access to high-quality green space is a clear disadvantage under the conditions of restrictions, not to mention the closure of social support structures that too often buffer urban poverty. As we are experiencing now, the crisis is aggravating existing injustices and runs the risk of leading to even larger injustices in the future. The longer restrictions endure, the larger the injustices may become. Thus, there is the need to move from exclusion to inclusion. So far, the core *Ambio* papers “avoid” being political, something that is difficult but necessary when studying diverse and contradictory urban systems and their societies. This is also necessary if we want to be successful in following Folke et al.’s core idea of creating and defending easy access to high-quality nature for all while respecting nature’s needs and supporting urban planning and policy-making. As scientists, we all face this *disciplinary blindness* of non-voluntary ignoring complementary knowledge and/or not being aware of methods of other disciplines (MacLeod 2018; Urbanska et al. 2019), and maybe it is something that cannot be solved by pushing the existing discipline but rather through interdisciplinary mixed-methods study that links, for example, urban ecology and political geography.
3. Social-ecological systems are places of inherent tension between their two dimensions, ecology and society. Thus, we need to rethink what we as urban ecologists mean by “co-evolution, co-habitation and co-existence” in urban systems. Non-human evolution in remnant wilderness systems—regardless of being situated in a city or beyond—has been continuously endangered by humans in a way that food webs and predator–prey relations were interrupted. As a consequence, cities were increasingly pushed into virgin ecosystems and thus multiplied contacts between humans and biodiversity. Such development provokes the crossover of pathogens in the form of zoonosis, which, similar to a boomerang effect, endangers humans, in particular in densely populated cities, and beyond. For the social-ecological systems and resilience of cities, this means sharing a larger habitat. Humans and wildlife require real niches in urban systems where wildlife can develop without any disturbance while being surrounded by buffer zones where access by humans and livestock is limited. Refraining from current increases in living space per urban capita, we must understand that it is the niche area for wildlife *in* and *around* cities that needs to increase first. The latter argument also supports biodiversity conservation at global scale and climate change mitigation.
4. Referring to our three *Ambio* papers, reconnecting humans (in cities) to the biosphere primarily means distancing them from distinct parts and areas of nature to keep biophysical processes and food webs alive and active and thus hinder pathogen jumps. We have considerable knowledge about the ranges of wildlife and diversities of healthy urban ecosystems in ecology. We must make use of them, and we have to understand that neither co-creation nor co-development is an all-encompassing and fully resilient stewardship of nature. If we include nature in what we call ‘co-creation’ and do not misunderstand it as an anthropocentric process but an equal one, we could reconnect to and co-create with nature. Having understood that co-evolution does not always mean co-habitation, rather partly parallel habitation and habitats that are spatially and functionally partly integrated, we will be able to create healthier and more resilient cities. Such cities offer space for humans, livestock and wildlife; they are embedded into larger urban-periurban and rural landscapes that respect wildlife and deliver the ecosystem benefits we seek: clean air, clean water, cooling and the beauty of plants and animals.
5. Finally, a recommendation for urban planning and design, based on the above points: We should strictly follow the idea of a real network of open spaces in cities and their peripheries that allow for both human outdoor stays—also in times of a pandemic—as well as safe outdoor life for wild animals. It seems mandatory to provide space for a healthy stay outside without crowding effects in a “fair way” and with whatever distancing is needed. Thus, we need clear limits for infill and densification in cities. We need space. When spatial resources are understood—at least in part—as a commons, values like affordable flats and houses, along with open green and blue spaces for humans and wildlife, should be as rewarding as any economic return rate. Equal accessibility of green and blue spaces for all would be prerequisites. The counterargument, urban sprawl, has been identified as extremely harmful to abiotic and biotic high-value nature around cities (McDonald et al. 2019). Thus, we need a novel paradigm of the inevitable dualism of densification and infill in cities that questions current and heads towards new design principles of densification, respecting the commons, wildlife, and fair access to green space. In this way, and relating back to the argument above, the human-used open space network does not interfere with the wildlife space but lives in proximity. Doing so, we allow wildlife in and around cities to find space to form stable biocoenosis, including all disease vectors that belong to it, and thus prevent the formation of zoonosis as best as we can while still allowing for people to move to cities.

Instead of a lengthy conclusion, I would propose that crisis is a Greek word, a fundamental one that allows for hybridity and different outputs. It is on us to stop developing global urbanization towards a catastrophe and instead to let it merge into a novel idea or ideology of consistent co-existence, nothing less than a profound reason and foundation for another 50 years of urban *Ambio* papers in the *Ambio* journal.
